# Cellular and Transcriptional Landscape of Human Hypoplastic Left Heart Syndrome

**DOI:** 10.21203/rs.3.rs-6689087/v1

**Published:** 2025-05-29

**Authors:** Kory Lavine, Farid Kadyrov, Junedh Amrute, Ivan Kuznetsov, Kristina Li, Zoltan Arany, Jonathan Edwards, Carmen Sucharov, Shelley Miyamoto

**Affiliations:** Washington University School of Medicine; Massachusetts General Hospital; Washington University School of Medicine; University of Pennsylvania; University of Pennsylvania; University of Pennsylvania; University of Pennsylvania; University of Colorado Anschutz Medical Campus; University of Colorado Anschutz Medical Campus

**Keywords:** Hypoplastic Left Heart Syndrome (HLHS), single-nucleus RNA sequencing (snRNA-seq) NRG3, CCN2, STAT3, endocardium, fibroblast

## Abstract

Hypoplastic left heart syndrome (HLHS) is a congenital heart defect characterized by impaired development of the left ventricle, often managed through surgical palliation creating a single ventricle (SV). Failure of the anatomical right ventricle (RV) represents a common complication with high mortality. We used single-nucleus RNA sequencing to generate a map of the pediatric non-failing (NF) and failing (SysHF) SV. Fibroblasts and endocardial cells displayed the greatest transcriptional shifts between NF and SysHF. Notably, activated fibroblasts expanded in SysHF, and endocardial cells in NF demonstrated adaptive transcriptomic shifts absent from controls or SysHF samples. Ligand-target analysis predicted disease-state specific signaling from endocardial cells to fibroblasts: NRG3 signaling in NF and CCN2 signaling in SysHF. *In silico* perturbation predicted *FOS, JUN,* and *STAT3* as regulators of fibroblast activation and endocardial adaptation. Finally, HLHS data was compared to adult human and murine RV failure to gain insight into shared pathological processes and the suitability of current animal models. These findings provide a comprehensive SV atlas and implicate cell non-autonomous signaling between endocardial cells and fibroblasts as drivers of SV systolic heart failure.

## Introduction

Hypoplastic left heart syndrome (HLHS) is among the most severe congenital heart diseases, and is characterized by underdevelopment of the left heart. Surgical palliation to generate a single-ventricle circulation that is dependent on anatomical right ventricle (RV) function represents a mainstay of treatment^1-3^. Despite ongoing advancements in surgical technique, single ventricle failure remains a common and serious complication that limits long-term survival^1,2,4,5^. The cellular and molecular drivers that govern single ventricle health and failure remain poorly understood and therapeutics that target single ventricle failure are yet to be developed. Notably, pediatric hearts exhibit substantial physiological and molecular differences from adult hearts, particularly under pathological conditions^6-8^. Consequently, therapeutic strategies developed for adult heart failure have shown limited efficacy in pediatric populations^1,5,9,10^.

Single-cell technologies enable high-resolution mapping of cardiac cell states, uncovering transcriptional cellular diversity in the context of disease^11,12^. Prior studies have utilized these techniques in healthy and diseased adult hearts to uncover the cellular landscape and map pathologic signaling mechanisms driving adult heart failure^6,13-22^. Few studies have explored congenital heart disease with a deep focus on single ventricle function^6^. Importantly, murine and adult human models have limitations in fully recapitulating congenital heart failure states^16^. Animal models, although invaluable for mechanistic insights, may fail to capture human-specific pathophysiological processes due to differences in heart development, ability to model the disease of interest, and temporal dynamics of disease progression^23-29^. Prior studies of human adult heart failure, driven primarily by acquired etiologies such as ischemic heart disease, hypertension, or metabolic stress, may not adequately represent congenital conditions driven by abnormal developmental trajectories and genetic predispositions^30-32^.

Many prior studies of single ventricle heart disease primarily relied on bulk transcriptomics, obscuring cell-type-specific discovery^7,8,33,34^. The emergence of single-nucleus RNA sequencing (snRNA-seq) offers an unprecedented opportunity to dissect cellular complexities at high resolution, enabling the identification of previously unrecognized disease-associated cell populations and regulatory networks. Previous studies have utilized snRNA-seq to profile small cohorts of congenital heart disease samples^6^ and hearts from individuals with genetic cardiomyopathies^21,22^, which has identified key drivers of pediatric heart failure. However, there is no controlled large-scale study which characterizes the cellular landscape in pediatric failing and non-failing single ventricles. An important challenge in pediatric heart disease is the scarcity of high-quality tissue especially in rare diseases such as HLHS that are linked to clinical outcomes. Such specimens are essential to provide insights into the molecular mechanisms driving disease progression in single ventricle patients with HLHS.

Herein, we performed snRNA-seq to construct a comprehensive cellular atlas of single ventricle HLHS hearts and age-matched non-diseased controls with a focus on determinants of systolic single ventricle failure. By integrating human pediatric single ventricle data with adult human RV failure, and murine RV failure model datasets, we uncovered pediatric-specific transcriptional signatures, defined cell-cell signaling pathways driving fibroblast activation and endocardial remodeling, and identified nodal transcription factors predicted to regulate single ventricle systolic failure. Our results provide novel insights into the unique molecular landscape of pediatric congenital heart failure, highlighting a cell non-autonomous signaling axis driving single ventricle systolic heart failure in HLHS.

## Results

### Cellular landscape of HLHS

**Figure 1:**
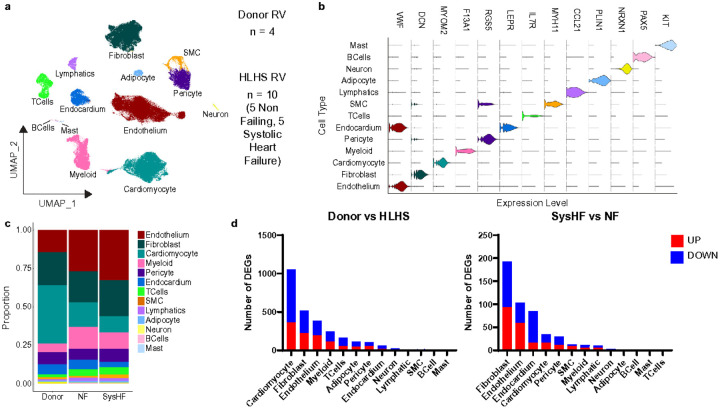
Global Clustering, Composition Analysis, and Differential Gene Expression of Right Ventricles from Donor and HLHS patients. a.) UMAP post QC of snSEQ data from n = 4 Donors, n = 5 Non-Failing (NF) HLHS, and n = 5 Systolic Heart Failure (SysHF) HLHS patients. b.) Violin plot showing canonical markers of identified sub populations. c.) Stacked bar graph showing the proportion of cell subclusters in Donor, NF and SysHF conditions. d.) Stacked bar graphs showing the number of upregulated (Red) and downregulated (Blue) genes when performing pseudobulk differential gene expression analysis between Donor and HLHS conditions (left) or between SysHF and NF conditions (right).

We performed snRNA-seq on transmural right ventricular tissue specimens from 4 non-diseased age-matched donors, 5 HLHS patients with preserved systolic function (NF), and 5 single ventricle HLHS patients with systolic heart failure (SysHF) (**Supplementary Table 1**). NF patients were selected for transplant because they had either protein losing enteropathy or plastic bronchitis. After quality control (Fig. 1a, **Supplementary Fig. 1**), we recovered 132,119 nuclei across 14 patients. Next, we performed dimensional reduction, integration, nearest neighbor clustering, uniform manifold approximation and projection (UMAP) construction, and cell clustering with differential gene expression to annotate 13 major cell types based on canonical marker genes (Fig. 1a-b). Notably, cell composition analysis showed expansion of immune cells (macrophages and T-cells) and endothelial cells in both failing and non-failing HLHS groups compared to donor (Fig. 1c). Pseudobulk DGE analyses revealed robust transcriptional differences between donor and HLHS hearts, as well as between NF and SysHF sub-groups (Fig. 1d). Notably, cardiomyocytes harbored the greatest transcriptional differences between donor and HLHS, consistent with prior studies comparing adult dilated cardiomyopathy (DCM) with non-failing donors. Interestingly, when we compared SysHF to NF within the HLHS group, we found that fibroblasts, endothelial cells, and the endocardium were encoded with the greatest transcriptional changes (Fig. 1d). Separation of the samples on a PCA plot on a per cluster basis validates the number of differentially expressed genes detected per cell cluster (**Supplementary Fig. 2**). These findings suggest that while cardiomyocytes transcriptionally shift in HLHS hearts similar to adult failure^16,19^, differences within the cardiac stroma are associated with single ventricle function in HLHS.

### Cardiac stromal cell diversification in HLHS

**Figure 2:**
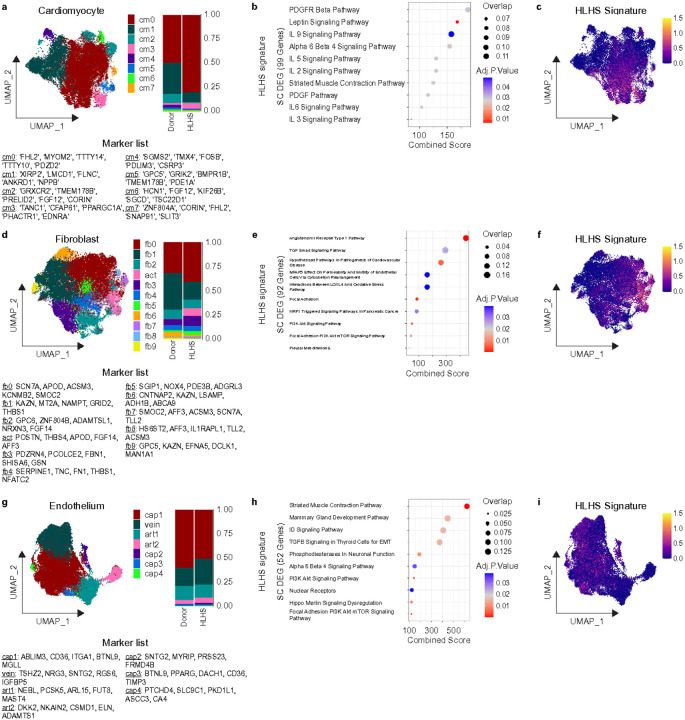
Subcluster Analysis of Cardiomyocytes, Fibroblasts, and Endothelium in Donor and HLHS conditions. a.) UMAP of identified cardiomyocyte subpopulations and stacked bar graph indicating the proportion of these subpopulations in Donor and HLHS conditions. b.) Wikipathways gene ontology analysis on genes up in HLHS when performing single cell differential expression analysis in comparison to donors. c.) The Z-scores of the HLHS signature (all genes upregulated in HLHS compared to Donor on the single cell level) of cardiomyocytes plotted on a UMAP projection. d.) UMAP of identified fibroblast subpopulations and stacked bar graph indicating the proportion of these subpopulations in Donor and HLHS conditions. e.) Wikipathways gene ontology analysis on the HLHS signature in fibroblasts. f.) The Z-scores of the HLHS signature of fibroblasts plotted on a UMAP projection. g.) UMAP of identified endothelium subpopulations and stacked bar graph indicating the proportion of these subpopulations in Donor and HLHS conditions. h.) Wikipathways gene ontology analysis on the HLHS signature in endothelium. i.) The Z-scores of the HLHS signature of endothelium plotted on a UMAP projection.

Given we saw the greatest transcriptional changes between donors and HLHS within cardiomyocytes, fibroblasts, and endothelial cells (Fig. 1d), we focused on these cell types to dissect differences in cell states between donors and HLHS hearts. We identified 8 transcriptionally distinct cardiomyocyte states (Fig. 2a, **Supplementary Fig. 3a-b**) and found that HLHS is associated with expansion of CM0 (*FHL2, MYOM2, TTTY14, TTTY10, PDZD2*) and a reduction in CM1 (*XIRP2, LMCD1, FLNC, ANKRD1, NPPB*) (Fig. 2a-b, **Supplementary Fig. 3a-b**). Notably, CM1 is enriched with genes classically increased in adult heart failure such as *ANKRD1* and *NPPB,* which highlights an important difference in myocyte cell state diversification between heart failure and single ventricle hearts. It has been previously shown that BNP expression is unchanged in HLHS RVs compared to non-failing pediatric RVs (). While NPPB expression is higher in Donor cardiomyocytes, the effect is diluted out when viewing the expression levels at the whole data level, in line with previously reported bulk mRNA data (**Supplementary Fig. 3c**). Differential gene expression on the single cell level (**Supplementary Fig. 3a**) and pathway analysis showed increased cytokine and growth factor signaling mediated by PDGFR, Leptin, IL2, IL3, IL5, IL6, and IL9 pathways in HLHS cardiomyocytes compared to donors (Fig. 2b). By overlaying the HLHS gene signature on the cardiomyocyte UMAP, we uncovered that these changes were mapped to CM0 and CM3 (Fig. 2c).

In the fibroblasts, we identified 11 transcriptionally distinct states (Fig. 2d, **Supplementary Fig. 3d-e**). Notably, we found an expansion of activated fibroblasts marked by *POSTN, THBS4, APOD, FGF14,* and *AFF3* (Fig. 2d, **Supplementary Fig. 3d-e**). Prior studies have shown that *POSTN* and *THBS4* expressing fibroblasts expand after myocardial infarction and persist in adult heart failure^16^. Differential gene expression at the single cell level (**Supplementary Fig. 3d**) and pathway analysis showed increased extracellular matrix remodeling, PI3K-AKT-mTOR signaling, and TGF *β* signaling in HLHS fibroblasts compared to donors (Fig. 2e). We then overlaid this gene signature on the fibroblast UMAP and identified that these genes were enriched in activated fibroblasts (Fig. 2f). Notably, within HLHS fibroblasts, we find increased expression of *LTBP2* (previously implicated in TGF *β* signaling, enriched in activated fibroblasts in adult heart failure, and is a serum biomarker for RV failure in pulmonary arterial hypertension patients.)^35,36^ and *IGFBP7* (previously implicated in pathogenic cancer associated fibroblasts)^37^. Collectively, these findings highlight that HLHS fibroblasts resemble a transcriptional state similar to what is observed in infarcted hearts, chronic heart failure, and cancer.

In the endothelium, we identified 7 transcriptionally distinct states (Fig. 2g, **Supplementary Fig. 3g**): 4 capillary, 2 arterial, and 1 venous endothelial state. HLHS specimens had a modest reduction in cap 1 (*ABLIM3, CD36, ITGA1, BTNL9, MGLL*) and an expansion in cap 2 (*SNTG2, MYRIP, PRSS23, FRMD4B*) (Fig. 2g). Differential gene expression at the single cell level (**Supplementary Fig. 3f**) and pathway analysis showed increased PI3K-AKT signaling and TGF *β* signaling associated with endothelial-to-mesenchymal (endoMT) transition (Fig. 2h, **Supplementary Fig. 3f**). The endothelial cell HLHS gene signature was enriched across multiple endothelial cell states (Fig. 2i). These findings highlight global transcriptional shifts across all endothelial cells in HLHS towards a transcriptional phenotype implicated in endoMT. However, there is still an overall increase in the number of endothelial cells in HLHS compared to donors (Fig. 1c).

### Inflammatory monocytes and macrophages expand in HLHS

Given the role of macrophages in coordinating the cardiac inflammatory response in heart failure, we sought to examine myeloid cell state changes between donor and HLHS hearts. We identified 11 transcriptionally distinct myeloid states (**Supplementary Fig. 4a-c**). Notably, we found an expansion in classical monocytes (mono1) and dendritic cells (DC1) and a reduction in mac 6 (*MAMDC2, SCN9A, F13A1*) in HLHS compared to donor (**Supplementary Fig. 4a**). Differential gene expression at the single cell level (**Supplementary Fig. 4d**) and pathway analysis showed enrichment of pathways implicated in autoimmune disease and allograft rejection in organ transplantation (**Supplementary Fig. 4e**). Interestingly, in HLHS we see increased expression of *NLRP3* which is part of the pro-inflammatory IL1 pathway and prior studies have showed expansion of *NLRP3* positive monocytes and macrophages in adult and genetic cardiomyopathies with spatial enrichment in areas of tissue damage and fibrosis^16,17,22^.

### Fibroblast expansion and activation are hallmarks of single ventricle systolic heart failure

**Figure 3:**
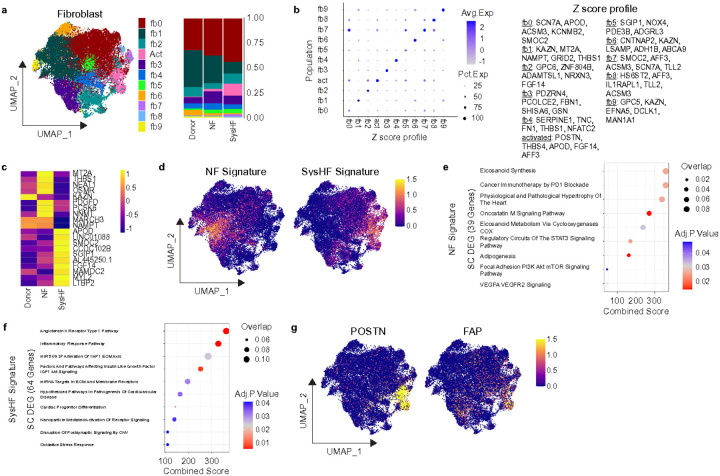
Activated fibroblasts expand in HLHS patients with systolic heart failure. a.) UMAP of subclustered fibroblasts and stacked bar graph indicating the proportion of these subpopulations in Donor, NF, and SysHF conditions. b.) Z-scores of signature genes from each subpopulation represented as a dot plot. c.) Heat map of the top 10 differentially upregulated and downregulated genes between NF and SysHF conditions on the single cell level. d.) The Z-scores of the NF and SysHF signatures (all genes upregulated in NF fibroblasts in comparison to SysHF and vice versa on the single cell level) of fibroblasts plotted on a UMAP projection. e.) gene ontology analysis of the NF signature in fibroblasts. f.) Wikipathway gene ontology analysis of the SysHF signature in fibroblasts. g.) Gene expression of POSTN and FAP in fibroblasts plotted on a UMAP.

In contrast to cardiomyocytes, fibroblasts showed marked transcriptomic shifts between NF and SysHF HLHS samples. To better characterize cell state shifts associated with NF and SysHF HLHS, we examined the distribution of fibroblast cell states (Fig. 3a-b). We observed a reduction in fb1 (*KAZN, MT2A, NAMPT, GRID2, THBS1*) and an expansion of activated fibroblasts (*POSTN, THBS4, APOD, FGF14, AFF3*) in SysHF compared to NF. Notably, there was a lesser expansion of activated fibroblasts in NF HLHS compared to donors highlighting that single ventricle physiology may induce transcriptional remodeling even in functionally preserved hearts (Fig. 3a). Differential gene expression analysis at the single cell level identified distinct transcriptional programs dysregulated in HLHS SysHF and NF compared to donors (Fig. 3c). We then overlaid the SysHF and NF gene signature in the UMAP space. Genes enriched in SysHF mapped to activated fibroblasts, while genes enriched in NF samples mapped to fb1 consistent with observed changes in cell state composition (Fig. 3d). Pathway analysis revealed enrichment of cytoskeletal and adhesion-associated genes in NF fibroblasts (Fig. 3e), while SysHF fibroblasts exhibited transcriptional signatures linked to inflammation and progression of cardiovascular disease (Fig. 3f). Notably, the activated fibroblasts in SysHF are enriched in *POSTN* and *FAP* which have been shown to expand following myocardial infarction and in chronic heart failure^16,17^, within cancer associated fibroblasts^38^, genetic cardiomyopathies^21,22^, and across fibrotic organs^38^. *FAP/POSTN^+^* fibroblasts have been causally linked to adverse cardiac remodeling^39^ and our findings highlight that therapeutic targeting of these pro-fibrotic programs may be relevant in single ventricle systolic heart failure.

### Endocardium in NF HLHS enters a state of adaptation

**Figure 4:**
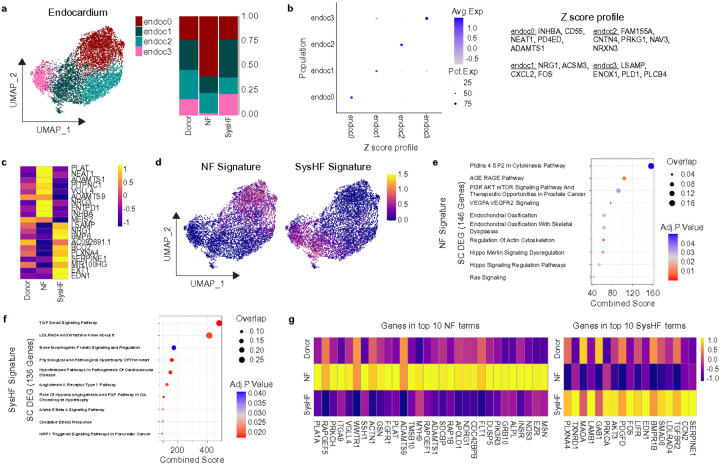
Endocardial cells differentially express signaling molecules in NF and SysHF conditions. a.) UMAP of subclustered endocardium and stacked bar graph indicating the proportion of these subpopulation in Donor, NF, and SysHF conditions. b.) Z-scores of signature genes from each subpopulation represented as a dot plot. c.) Heat map of the top 10 differentially upregulated and downregulated genes between NF and SysHF conditions on the single cell level. d.) The Z-scores of the NF and SysHF signatures (all genes upregulated in NF endocardium in comparison to SysHF and vice versa on the single cell level) of endocardium plotted on a UMAP projection. e.) Wikipathway gene ontology analysis of the NF signature in endocardium. f.) Wikipathway gene ontology analysis of the SysHF signature in endocardium. g.) Heatmap of the genes that make up the top 10 gene ontology terms in NF and SysHF conditions.

We identified 4 transcriptionally distinct endocardial cell states (Fig. 4a-b) and detected expansion of endoc0 (*INHBA, CD55, NEAT1, PD4ED, ADAMTS1*) and reductions in endoc3 (LSAMP, ENOX1, PLD1, PLCB4) and endoc1 (*NRG1, ACSM3, CXCL2, FOS*) in NF relative to SysHF specimens (Fig. 4a). Differential gene expression analysis at the single cell level between NF and SysHF groups revealed distinct transcriptional programs enriched in SysHF and NF compared to donor controls (Fig. 4c). We then overlaid the SysHF and NF gene signatures in UMAP space and found enrichment of the SysHF signature in endoc1 and endoc3 and enrichment of the NF signature in endoc0, consistent with the cell state compositional changes (Fig. 4d). Pathway analysis showed that NF endocardium displayed angiogenic and cytoskeletal gene expression, while SysHF endocardium was enriched for TGFp and BMP signaling (Fig. 4e-f). Next, we generated heatmaps of the genes present in the top NF and SysHF terms (Fig. 4g). Notably, we found that genes upregulated in NF compared to SysHF were minimally expressed in donor endocardial cells suggesting that the NF may represent an adaptive phenotypic state not seen in a healthy heart. Interestingly, *FLT1* and *NOS3*are increased in NF relative to SysHF and donor (Fig. 4g). These findings highlight potential involvement of angiogenic pathways and bolster the concept that maintenance of single ventricle function in HLHS may involve a state of compensation analogous to findings in left ventricular recovery seen after mechanical unloading^15,40^. Several genes increased in SysHF including *PDGFD, FOS, EDN1, SMAD6, TGFBR2, CCN2,* and *SERPINE1* have been previously implicated in pro-fibrotic remodeling^41^. In addition, many genes dysregulated in NF and SysHF conditions were secreted ligands: *CCN2, EDN1, PDGFD, NRG3, INHBA, NRG1,* and *BMP6,* implying that the endocardium may be an important contributor to cell autonomous and non-autonomous signaling in these disease states.

### Cell non-autonomous signaling in HLHS drives regulatory programs

**Figure 5:**
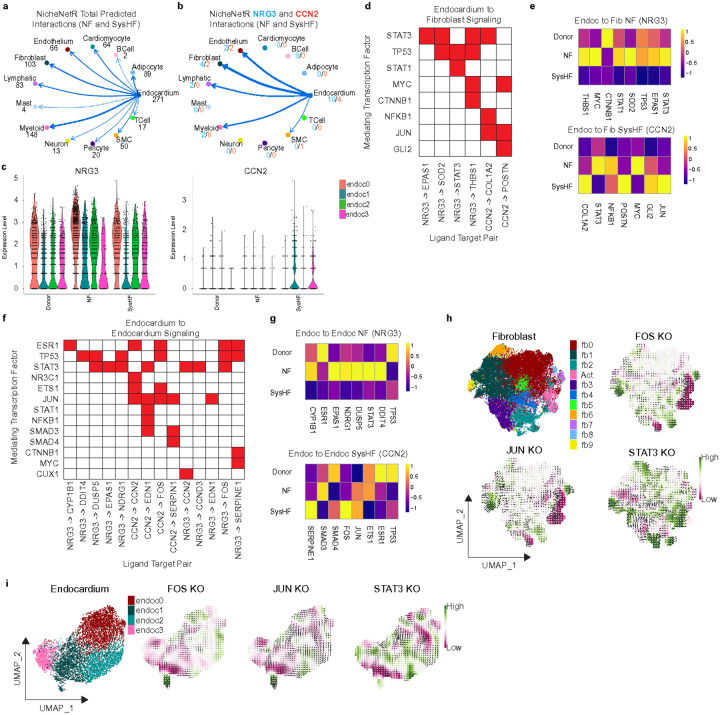
Ligand target analysis and *in silico* perturbation reveals differential NRG3 and CCN2 signaling. a.) NicheNetR analysis using endocardium as a source and every cell type (including endocardium) as the receiving cell type. Numbers indicate the total number of detected interactions. b.) Total number of interactions detected by NicheNetR using NRG3 (blue) or CCN2 (red) from endocardium as a source. c.) Violin plot of *NRG3* and *CCN2*expression in Donor/NF/SysHF endocardium split by endocardial subpopulation. d.) Binary heat map indicating the transcription factors (y axis) mediating NRG3 or CCN2 ligand target interactions between endocardium and fibroblast as detected by NicheNetR. Red indicates that the transcription factor is mediating the ligand target interaction. e.) Heat map of the genes in d indicating their expression in Donor, NF, and SysHF conditions. f.) Binary heat map indicating the TFs mediating NRG3 or CCN2 ligand target interactions in autonomous endocardium signaling. g.) Heat map of the genes in f indicating their expression in Donor, NF, and SysHF conditions. h.) UMAP of subclustered fibroblasts and their annotation, and CellOracle in silico KO simulation perturbation scores and overlaid quiver plot in fibroblasts displayed on a UMAP. Red indicates less likely to differentiate and green indicates more likely to differentiate. i.) UMAP of subclustered endocardium and their annotation and CellOracle in silico KO simulation perturbation scores and overlaid quiver plot.

To explore predicted cell-cell communication events in HLHS (NF vs. SysHF), we performed un-biased ligand-target inference using NicheNetR^42^ which suggested prominent interactions between endocardial cells and fibroblasts (Fig. 5a, **Supplementary Fig. 5**). We focused on fibroblasts as those had the most differences between SysHF and NF (Fig. 1d). This analysis indicated that NRG3 signaling from endocardium to fibroblasts was enriched in NF HLHS samples. Conversely, CCN2 signaling from endocardium to fibroblasts was enriched in SysHF HLHS samples (Fig. 5b, **Supplementary Fig. 5**). Interestingly, NRG3 and CCN2 were predicted to interact with receptor specific downstream transcription factor modules in NF and SysHF HLHS samples in fibroblasts. In NF endocardial cells, NRG3 was inferred to activate STAT1, STAT3, MYC, TP53, and CTNNB1 through NRG3-EPAS1, NRG2-SOD2, and NRG3-THBS1 signaling (Fig. 5c-d). In SysHF endocardial cells, *CCN2* was predicted to activate GLI2, JUN, MYC, NFKB1, STAT3, and JUN in fibroblasts via CCN2-COL1A2 and CCN2-POSTN signaling (Fig. 5c-d). Interestingly, NRG3 autocrine signaling in NF and CCN2 autocrine signaling in SysHF within endocardial cells was inferred to engage distinct and shared transcriptional modules (Fig. 5e-f, **Supplementary Fig. 5**).

### Network based prioritization of transcriptional targets

To elucidate mechanisms that underlie fibroblast cell state transitions in HLHS, we applied pseudotime trajectory analysis and overlaid transcription factor module expression and inferred activity in UMAP space (**Supplementary Figure. 6a-c**). We found that *FOS* and *JUN* expression and activity were increased along the activated fibroblast trajectory, while *STAT3* was enriched within quiescent fibroblasts (**Supplementary Figure. 6a-c**). *In silico* transcription factor perturbation revealed that simulated deletion of *FOS* and *JUN* moved fibroblasts away from the activated state, which was enriched in SysHF. In contrast, simulated deletion of *STAT3* accelerated transition into the activated fibroblast cell state (Fig. 5g). To examine cell state transitions in endocardial cells in HLHS, we performed pseudotime trajectory analysis and overlaid transcription factor module expression and activity in UMAP space (**Supplementary Fig. 6d-f**). In silico transcription factor perturbation showed that simulated deletion of *FOS, JUN,* and *STAT3* led to a shift away from endoc1 and endoc3, which were enriched in SysHF (Fig. 5h). These findings highlight specific signaling pathways and transcriptional events that may prevent endocardial cell phenotypic shifts, fibroblast activation and resultant fibrosis observed in single ventricular systolic failure. Confirmation of these putative targets requires functional validation in suitable experimental models.

### HLHS is transcriptionally distinct from adult and murine RV heart failure

Identification of appropriate model systems is necessary to further investigate the relevance of disease-associated signaling pathways identified in our HLHS snRNA-seq data. As such, we sought to determine the feasibility of utilizing a common murine model of right ventricle failure (RVF), pulmonary artery banding (PAB) (INSERT CO-SUBMISSION REFERENCE HERE). snRNA-seq was performed two weeks after PAB. Animals were separated into normal (norm.) and 2 groups of PAB: moderate (mod.) and severe (sev.) RVF groups were based on whether RV end-diastolic area had increased by less than or greater than two-fold, respectively. Mouse snRNA-seq data from the PAB model were analyzed using the same pipeline applied to the HLHS data, followed by reference mapping onto the HLHS dataset using Seurat’s label transfer method (**Supplementary Figure. 7a**).

Composition analysis of predicted cell types revealed that the mouse PAB model recapitulated some, but not all, cellular features of HLHS (**Supplementary Figure. 7b**). Similar to HLHS, PAB mice displayed increased endothelial cells and reduced cardiomyocyte proportions. However, unlike HLHS, PAB mice did not exhibit increased myeloid cells and displayed a pronounced increase in fibroblast populations (Fig. 1c, **Supplementary Figure. 7b**). The accuracy of the reference mapping was assessed by mapping scores, which indicated strong correspondence between mouse and HLHS endothelial cells, fibroblasts, cardiomyocytes, myeloid cells, T cells, and lymphatic cells. Mapping of pericytes, endocardial cells, smooth muscle cells (SMCs), adipocytes, neurons, B cells, and mast cells showed less substantial correlation (**Supplementary Figure. 7c**).

**Figure 6:**
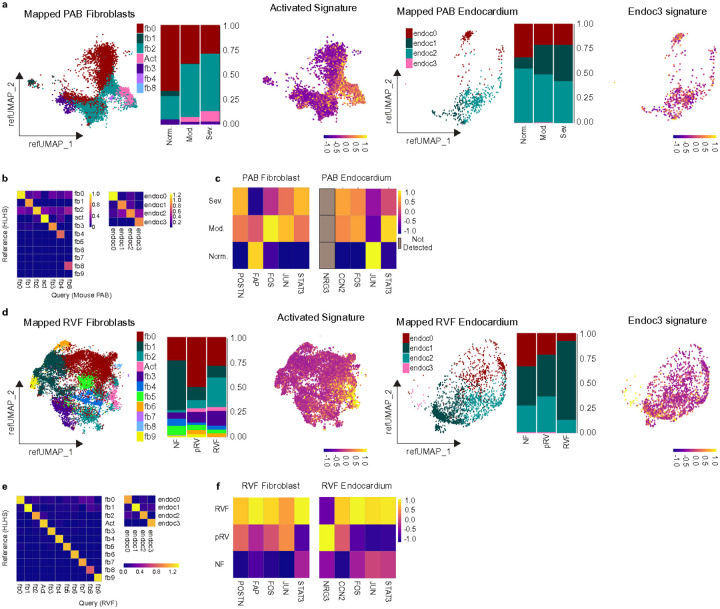
Mouse PA banding and Adult Human RV failure recapitulates some but not all aspects of HLHS. a.) Reference UMAP of mouse PA banding (n = 3 normal, n = 3 moderate, n = 4 severe) fibroblasts and endocardium mapped to HLHS data. Stacked bar graphs show the proportion of predicted cell types in normal, moderate, and severe conditions. b.) Heat map showing the mapping scores when mapping the Query (PA banding fibroblasts/endocardium) to the reference (HLHS fibroblasts/endocardium). c.) Average SCT expression per sample of *Postn* in fibroblasts and *Ccn2* in endocardium in normal, moderate, and severe PA banding mice. d.) Reference UMAP of fibroblasts and endocardium from RVF data when mapped to HLHS data, and stacked bar graphs representing the proportion of predicted fibroblast and endocardium cell types in NF, pRV, and RVF conditions. e.) Heat map showing the mapping scores when mapping the Query (RVF fibroblast or endocardium subpopulations) on the Reference (HLHS fibroblast or endocardium subpopulations). f.) Heat map of genes of interest in RVF fibroblast and endocardium data.

Further analysis of fibroblast and endocardial subclusters via reference mapping identified distinct patterns (Fig. 6a-c). Fibroblast populations in PAB mice mirrored several changes observed in HLHS. Activated fibroblasts mapped effectively between datasets and showed similar expansion trends in PAB versus sham mice, analogous to their expansion in HLHS compared to donor hearts (Fig. 6a-b). The signature of activated fibroblasts from HLHS data fell in line with predicted cell types in PAB data (Fig. 6a). Moreover, the expression of *Postn* was significantly elevated in PAB mice compared to sham controls (Fig. 6c). Additionally, fibroblast signatures from NF and SysHF HLHS datasets demonstrated correlations between sham mice and the NF signature, whereas PAB mice correlated with the SysHF and HLHS signatures (**Supplementary Figure. 7d**). Conversely, endocardium from the PAB model poorly reflected HLHS-specific observations. The subpopulation endoc3 was notably underrepresented in the PAB dataset, and effective mapping was restricted mainly to the endoc0 subcluster (Fig. 6a-b). The signature of endoc3 from HLHS data did not bias itself to any particular cell cluster (Fig. 6a). *Nrg3* was not detected in PAB data (Fig. 6c), and correlations of endocardial NF, SysHF, and HLHS signatures with PAB conditions were not evident (**Supplementary Figure. 7e**).

To evaluate whether human adult RV failure recapitulated cell state and transcriptional shifts observed in HLHS, we mapped human adult RVF datasets onto our HLHS single-cell atlas using the same strategy as above (**Supplementary Fig. 8a-c**) (REFERENCE TO COSUBMISSION ARTICLE). This snRNA-seq dataset was generated from human RV specimens obtained from non-diseased donors (NF) and diseased RV from dilated cardiomyopathy that was divided into groups: preserved RV function (pRV) and failing RV (RVF). Composition analysis revealed that proportion of predicted cell types in adult RVF reflected many aspects of HLHS on the global level. There was an increase in endothelium, a decrease in cardiomyocytes, and an increase in myeloid cell types in RVF and pRV in comparison to NF (**Supplementary Fig. 8c**). Mapping scores indicated high correspondence between all cell types in RVF and HLHS data except B cells and Mast cells, which were present in small numbers (**Supplementary Fig. 8d**).

However, subcluster analysis of mapped fibroblasts and endocardium revealed both similarities and differences between HLHS and adult RVF. There was a modest increase in activated fibroblasts in RVF data and an underrepresentation of endocardial cell type endoc3, although endoc3 was detected unlike mouse PAB data (Fig. 6d-e). Activated fibroblast and endoc3 signatures plotted on endoc3 reflected this concept (Fig. 6d). Fibroblast HLHS signatures correlated with fibroblast RVF conditions, however endocardium HLHS signatures did not correlate between NF, pRV, and RVF conditions (**Supplementary Fig. 8e**). Similar to SysHF HLHS fibroblasts, RVF fibroblasts displayed enriched levels of *POSTN, FAP, FOS,* and *JUN,* consistent with a preserved activated fibroblast population observed across human heart failure etiologies. In contrast, RVF fibroblasts contained higher expression of STAT3, which was not observed in SysHF HLHS fibroblasts. pRV endocardium expressed higher levels of *NRG3* and RVF endocardium expressed *CCN2, FOS,* and *JUN* similar to what was seen in HLHS when comparing NF to SysHF. However, RVF endocardium expressed higher levels of *STAT3,* which was evident in NF HLHS endocardium (Fig. 6f). These findings highlight shared and distinct features of RV failure between single ventricle and biventricular hearts at the transcriptional level.

## Discussion

Advances in single cell multiomics have paved the way for a human first approach to discovery. Within the cardiovascular field, these techniques have provided new insights into heart failure pathology where they have uncovered cell states that drive adverse remodeling^13-22^. Recent studies in congenital heart disease and genetic cardiomyopathies have shed light on crucial differences between adult and pediatric heart failure^6,21,22^. To date, there is no comprehensive transcriptomic atlas of pediatric HLHS, and little is known regarding the molecular mechanisms which drive systolic decompensation in this disease. Herein, we perform snRNA-seq in pediatric donor controls and pediatric single ventricle specimens obtained from HLHS patients with normal (NF) or reduced systolic function (SysHF) to construct a comprehensive map of pediatric single ventricle heart disease. Using integrated analyses, we find that stromal cells harbor the greatest transcriptional differences between normally functioning and failing single ventricle and identify signaling and transcriptional mechanisms that may underpin systolic decompensation.

Numerous studies have established that cardiac cell types acquire disease associated states in the setting of adult and congenital heart disease^13,14,19^. We observed the most profound transcriptional changes between donor and HLHS hearts within the cardiomyocytes, fibroblasts, and endothelial cells (consistent with prior work in adult heart failure). Among these populations, we found that fibroblasts and endocardial cells harbored the greatest transcriptional differences between NF HLHS and SysHF HLHS.

Recent studies have uncovered a prominent role for activated fibroblasts that express *FAP* and *POSTN* as a key driver of fibrotic remodeling in infarcted hearts and chronic forms of adult heart failure^16,43,44^. Prior studies have profiled pediatric dilated cardiomyopathy hearts with known genetic mutations and shown expansion of *FAP^+^* activated fibroblasts in areas of active inflammation and fibrosis^21,22^. More recent work characterized 3 HLHS pediatric hearts and showed increased fibrosis by trichrome staining^6^. Here we characterize the fibroblast cell diversity in donors, NF HLHS, and SysHF HLHS – notably, we found a profound expansion of *POSTN^+^FAP^+^* fibroblasts in SysHF samples relative to donors and NF HLHS. These findings highlight the absence of this pathogenic population in single ventricle hearts with preserved systolic function and an expansion in the setting of systolic decompensation, potentially implicating fibrosis as a pathologic mechanism of single ventricle failure. Outside of pathological analysis, there are additional opportunities to explore the contribution of fibrosis in single ventricle patients including non-invasive PET imaging to identify FAP uptake in human and mouse disease^16,39,45-47^, which could serve as a surrogate feature and provide insight on patient selection for preventative therapy. Previous work has also shown fibrosis to not be a major contributor to RV failure in pediatric subjects with a single ventricle (70). It is possible that there is no increase in fibrosis, rather fibroblasts take on a pathogenic identity. This pathogenic gene signature may have been diluted out in previous whole RNA approaches but is detected with single nuclei approaches. It is also possible that endocardial signaling to other cell types drives RV pathogenesis in pediatric SV.

Interestingly, we observed that endocardial cells take on differing fates in NF and SysHF conditions underscoring the pivotal role of endocardial remodeling in single ventricle systolic decompensation^48^. Strikingly, NF HLHS endocardial cells appeared to acquire a state distinct from donor and SysHF endocardial cells, indicating a state of adaptation or compensation. NF HLHS endocardial cells expressed high levels of *FLT1* suggesting a potential pro-angiogenic fate that contributes to beneficial remodeling of the single ventricle. Given the role of *FLT1* in angiogenesis and vascular development^49-54^ these findings posit the possibility that dysregulation of this pathway within the endocardium may contribute to the progression of HLHS. These findings parallel prior work in adult cardiac recovery where recovered cell states take on an adaptive phenotype not found in a healthy heart^15^.

Numerous studies have shown that organ dysfunction particularly in the heart is mediated through cell non-autonomous signaling events mediating stromal cell state transitions^16,55^. Recent work in adult myocardial infarction and chronic heart failure uncovered macrophage-fibroblast crosstalk as the key driver of cardiac remodeling^16,55^. Here, we used unbiased cell-cell signaling analysis and network-based prioritization to uncover a unique HLHS-specific signaling axis not previously described in adult or murine heart failure models. We found a NRG3 signaling axis between endocardial cells and fibroblasts via STAT3 was enriched in NF samples. Conversely, we found a CCN2-driven signaling axis between endocardial cells and fibroblast was enriched in SysHF. These findings also highlighted potential contributions of endocardial cells as drivers of pro-fibrotic cell state transitions through JUN and FOS activation. Additionally, we used network-based prioritization and in silico perturbation analysis to show that targeting *STAT3, FOS,* and *JUN* in silico may shift endocardial and fibroblast cell states away from SysHF towards NF HLHS states.

A key challenge in studying HLHS is the lack of validated human *in vitro* and animal *in vivo* model systems that recapitulate the human phenotype. To explore this, we compared our human HLHS data to a murine model of PAB and RV failure. We found that that the failing mouse RV recapitulated signatures of pro-fibrotic remodeling with an expansion of *FAP/POSTN^+^* fibroblasts found in SysHF HLHS hearts. However, the mouse RV failure model failed to recapitulate endocardial cell states found in pediatric HLHS samples, which underscores the limitations of existing animal models. Additionally, these findings support the notion that endocardial cells in HLHS may be a driver population in initiation and progression of the single ventricle disease development. It will be informative to understand whether neonatal PAB models may serve as a superior model. Similarly, we integrated our data with a dataset generated from biventricular patients with RV failure and found overlapping cell states within the fibroblast compartment. SysHF HLHS and adult RV failure^56^ showed expansion of *FAP/POSTN^+^* fibroblasts with enriched *CCN2* mediated signaling from endocardium. However, there was little overlap within the endocardial cell states. These findings highlight the broader conservation of pathogenic fibroblast population in driving adverse cardiac remodeling across different heart failure etiologies, age ranges, and disease contexts^16,38^. Collectively, these findings highlight the importance of developmental context as a critical determinant of disease mechanisms.

Our study is not without limitations. The relatively small patient sample size may limit the generalizability of our results, and future studies involving larger cohorts would strengthen these observations. Additionally, the cross-sectional nature of our study precludes longitudinal insights into the temporal dynamics of cellular transitions and disease progression across the varied pathophysiologies experienced during HLHS staged palliation. Finally, functional validation of identified signaling pathways and transcription factors in *in vitro* and *in vivo* remains necessary to confirm their therapeutic potential. A key challenge with downstream validation is the lack of tool systems which recapitulate the human HLHS phenotype.

In conclusion, we generate a comprehensive human HLHS cell atlas and uncover unique cell non-autonomous signaling events between endocardial cells and fibroblasts mediating systolic decompensation in HLHS hearts. Furthermore, we integrate cell-cell signaling analysis with network-based prioritization to prioritize potential network correcting targets which can facilitate reversion of SysHF into NF HLHS cell states. Collectively, our findings highlight the crucial similarities and differences between pediatric HLHS and adult heart failure and prioritize cell types and molecular pathways driving single ventricle systolic decompensation.

## Methods

### Ethical approval for human specimens

This study complies with ethical regulations for human research and was approved by the Washington University institutional review board 201104172. All samples were procured with informed consent. Demographic details are available in **Supplementary Table 1**.

### Single nuclei sample preparation

Single nuclei suspensions were generated as previously described^15,19,22^. In brief: flash frozen sections were minced with a razor blade, transferred to a Dounce Homogenizer containing 1 mL of lysis buffer (10 mM Tris-HCl, pH 7.4, 10 mM NaCl, 3 mM MgCl_2_ and 0.1% NP-40 in nuclease-free water) on ice. Samples were homogenized using five strokes, an additional 1 mL of lysis buffer added, and incubated on ice for 15 mins. Samples were then filtered with a 40μm filter and filter was rinsed with 1mL of lysis buffer. The mixture was then centrifuged at 500*g* for 5 min 4 °C, resuspended in 1mL nuclei wash buffer (2% BSA and 0.2 U μl^−1^ RNase inhibitor (Thermo Fisher, cat. no. AM2694) in 1× PBS) and, filtered using a 20*μ*m pluristrainer (Pluriselect, cat. No. SKU43-50020-03). Filtered solution as centrifuged using the above criteria and resuspended in 300 μL Nuclei Wash Buffer and transferred into a 5mL tube for flow cytometry. Subsequently, 1 μl DRAQ5 (5 mM solution; Thermo Fisher, cat. no. 62251) was added, sample gently vortexed, and allowed to incubate for 5 min prior to sorting. DRAQ5^+^ nuclei were sorted into 300 μL Nuclei Wash Buffer using a BD FACS Melody (BD Biosciences) with a 100 μM nozzle. Sorted nuclei were then centrifuged using the above conditions and resuspended in Nuclei Wash Buffer for a final target concentration of 1,000 nuclei/μL – nuclei were counted on a hemocytometer. Based on the nuclei concentration, 10,000 target nuclei were loaded onto a Chip K for GEM generation using the Chromium Single Cell 5 Reagent v2 kit from 10X Genomics. Reverse transcription, barcoding, complementary DNA amplification and purification for library preparation were performed as per the Chromium 5 v2 protocol at the McDonnel Genome Institute. Sequencing was performed on a NovaSeq 6000 platform (Illumina) at a target read depth of 50,000 at the McDonnel Genome Institute.

### Generation of global object

FastQ files were aligned to the GRCh38-2020-A transcriptome using 10x Genomics Cell Ranger version 6.1.1. Filtered feature matrices from Cell Ranger were loaded into Seurat v 4.4.0^57-59^ and QC filters of nuclei with greater than 1000 and less than 10000 read counts and less than 5 percent proportion of mitochondrial genes were applied. The objects were then processed by scrublet^60^ version 0.2.3 to detect and remove doublets. Nuclei with a scrublet score of greater than 0.25 were excluded from further analysis. The object was then normalized with Seurat using SCTransform^61^ regressing out the mitochondrial percentage and RNA read counts. PCA was then calculated, followed by Harmony^62^ v1.2.0 integration using each sample as the covariate, and then a UMAP embedding was constructed. FindNeighbors, FindClusters, and FindAllMarkers was run in order to cluster the data and find genes that are differentially expressed in each cluster. FindAllMarkers used the Wilcoxon Rank Sum test with cutoffs of a minimum fraction of cells of 0.1 and a log fold change threshold of 0.25. A cell type was assigned to each cluster based on the genes they expressed from FindAllMarkers. Cell type assignment was performed manually and was informed by canonical expression of cell type markers.

For further QC and cleaning of the object, each major cell type: cardiomyocyte, endocardium, endothelium, fibroblast, myeloid, smc, pericyte, tcells, lymphatic, adipocyte, neuron, bcell, and mast were individually subset and then reclustered using the same SCTransform, PCA, Harmony, UMAP, FindNeighbors, FindClusters, and FindAllMarkers parameters as for the global object. This allowed for the identification and removal of nuclei that did not express genes of their identified cell type and/or nuclei that had expression of multiple cell types, which are assumed to be doublets or low quality nuclei. This process of subsetting, re-normalizing, re-integrating, re-clustering, and re-annotating was repeated until all doublet and low quality nuclei were removed.

These cleaned cardiomyocyte, endocardium, endothelium, fibroblast, myeloid, smc, pericyte, tcells, lymphatic, adipocyte, neuron, bcell, and mast objects were then merged, re-normalized, re-integrated, and re-clustered as above for a final global snSEQ object. Annotations from the original clustering were retained, and accuracy was double checked by generating a violin plot using the Seurat function VlnPlot for canonical cell type markers.

### Pseudobulk differential gene expression analysis

Using the cleaned global object, the RNA counts of each nuclei were extracted, and then aggregated based on the global cell type annotation. DESeq2^63^ v1.40.2 was used to perform differential gene expression analysis between conditions on a per cell type bases on the aggregated RNA expression data. An adjusted p value cutoff of 0.05 was used. Due to the high ambient expression of cardiomyocyte RNA, cardiomyocyte specific genes were “censored” from the differentially expressed gene lists in all non cardiomyocyte cell types. This was accomplished by performing FindAllMarkers on the cleaned global object on each cell type, identifying genes with a > 1 log fold change expression in cardiomyocytes, and removing those identified genes from each differentially expressed gene list.

### Subcluster analysis

Subcluster analysis of cardiomyocytes, fibroblasts, endothelium, endocardium, and myeloid cells used the cleaned subcluster objects that were generated prior to generation of the final global object. Subclusters were annotated manually based on genes specifically expressed in subclusters identified by the FindAllMarkers function. Specific expression of the marker lists was ensured by calculating a Z-score of identified marker genes and plotting them as a dot plot across each subpopulation using the Seurat function DotPlot. For fibroblasts, activated fibroblasts were manually subset by calculating a z-score of each cell based on their expression of POSTN and THBS4, and calling any cell that had a Z-score of this signature greater than 1.5 as a “activated fibroblast.”

For downstream gene ontology, heatmaps, and condition signature analysis, gene lists from differential expression on the single cell level was used instead of pseudobulk. This was accomplished by running FindMarkers in Seurat on the cleaned global object on each cell subpopulation. A log fold change cutoff of greater than 0.4 or less than −0.4 and an adjusted p value cutoff of 0.5 was applied. Cardiomyocyte censoring was performed on these differentially expressed gene lists as described in pseudobulk differential gene expression analysis.

“Signatures” for each condition are Z-scores calculated for all genes upregulated in that condition, and plotted on a UMAP in Seurat. Gene ontology analysis was performed through EnrichR using the WikiPathway 2023 Human data set. Only pathways with an adjusted p value less than 0.05 were considered. Heatmaps were generated by using the AverageExpression function in Seurat for a gene using the SCT assay, and plotting it using pheatmap version 1.0.12.

### Receptor ligand analysis and transcription factor enrichment

NicheNetR^42,64,65^ version 2.0.4 was used to perform receptor ligand analysis on the cleaned global HLHS object using endocardium as the sending cell type. For condition specificity, the “condition of interest” was set to either NF or SysHF. To stay in line with previous analyses, differentially expressed genes used in the NicheNetR pipeline excluded genes with a adjusted p value greater than 0.05 and a log fold change less than 0.4 or greater than −0.4. For visualization purposes, ligands that were also in the target column were not represented on circos plots. Circos plots were generated using the chordDiagram function in circlize version 0.4.16. Transcription factors mediating receptor target interactions of interest were identified in NicheNetR using the get_ligand_signaling_path function. Transcription factor activity scores for transcription factors of interest were generated using the decoupleR^66^ package. The CollecTRI^67-69^ gene regulatory network was used to determine the transcription factor activity, and these values were plotted on the UMAP projection.

### Pseudotime and in silico perturbation

To prepare snSEQ data for in silico perturbation, Seurat objects of the endocardium and fibroblast subclusters were converted to h5ad format using MuDataSeurat. The starting cell for trajectory analysis was determined by finding the cell with the highest Donor signature via Z-score. Palantir version 1.0.0 was used to assign pseudotime scores to each cell. Default settings were used to determine the number of eigenvalues. The number of waypoints selected was 500. The option “use_early_cell_as_start” was set to true. The data was then analyzed with Cell Oracle^69^ to construct a gene regulatory network (GRN) and perform in silico perturbations. Instead of down sampling the data and only looking at the top 2000-3000 highly variable genes, the entire dataset was used. Instead of using the base GRN, a transcription factor target gene pair dictionary was generated from information available from the CollecTRI database, but excluding negative regulation. In silico perturbation was then performed using Cell Oracle. Scale parameters, grid points, min_mass, and vm parameters were adjusted using the heuristics described in the Cell Oracle vignette.

### Reference mapping

RVF and mouse PA banding data was acquired from INSERT CO-SUBMISSION HERE. Data was reanalyzed exactly as was performed for HLHS data. To compare mouse PA banding data to HLHS, the gene names in the Seurat objects were first converted to using gprofiler (https://biit.cs.ut.ee/gprofiler/orth). It is important to note that not every mouse gene had a human ortholog. To compare RVF and PA banding data to HLHS, reference mapping was performed using FindTransferAnchors and MapQuery in Seurat, in order to predict what cell type RVF and PA nuclei would be in the HLHS data and to project the data onto the HLHS UMAP.

### Statistics and Reproducibility

No sample size calculations were performed. Sample size was governed by tissue availability and input tissue mass was based on ability to recover sufficient nuclei. No samples were excluded. For human studies all samples with HLHS NF, HLHS SysHF, and non-failing donors were processed randomized across age, sex, and race. Blinding during data collection was not necessary as nuclei isolation protocol required FACS to collect intact nuclei with no exclusion of any cells/nuclei. This sorting approach does not introduce any bias into the sample collection.

## Data Availability

Data will be available upon publication at NCBI GEO.

## Code availability

Code will be available upon publication at https://github.com/fkadyrov/hlhs.
